# Tetrandrine Inhibits the Wnt/****β****-Catenin Signalling Pathway and Alleviates Osteoarthritis: An In Vitro and In Vivo Study

**DOI:** 10.1155/2013/809579

**Published:** 2013-03-05

**Authors:** Xindie Zhou, Weijun Li, Lifeng Jiang, Jiapeng Bao, Lijiang Tao, Jin Li, Lidong Wu

**Affiliations:** Department of Orthopedics Surgery, The Second Affiliated Hospital of Medical College, Zhejiang University, Hangzhou 310000, China

## Abstract

There is currently no effective drug treatment for the early phase of osteoarthritis (OA), one of the most common senile diseases. The goal of this study was to investigate the protective effect of the tetrandrine (Tet) on OA, in vitro and in vivo. In an in vitro experiment, quantitative real-time polymerase chain reaction (qRT-PCR) was used to investigate changes in gene expression upon the addition of Tet in chondrocytes processed with IL-1**β**; changes in protein profiles were assessed by Western blotting. In vivo, to determine whether Tet has the protective effects on articular cartilage, a rabbit anterior cruciate ligament transaction model of OA was established. Expression of matrix metalloproteinase and **β**-catenin genes increased significantly, while that of tissue inhibitor of metalloproteinase-1 decreased significantly in the OA group both in vivo and in chondrocytes. However, the changes of expression were reversed by Tet, and there was less cartilage degradation in vivo compared with the OA group, as assessed by histological and macroscopic observations. Thus, Tet may play a useful role in the treatment of OA through the Wnt/**β**-catenin signalling pathway and has potential for the treatment of OA.

## 1. Introduction

Osteoarthritis (OA) is the most common joint disorder and is a major cause of pain, disability, and loss of quality of life. It is regarded as a degenerative disease and is associated with joint marginal osteophyte formation. It is believed that obesity, bone mass, joint injury and instability, developmental diseases, trauma, joint deformity, and age are common factors in OA, especially of the hip and knee joints [1]. Although OA is regarded primarily as a noninflammatory arthropathy, symptoms of local inflammation, as well as synovitis, are present in many patients and animal models of OA during cartilage destruction [2]. Interleukin-1 (IL-1), originally known as a lymphokine, was first described in the context of cellular interactions in articular tissues as a monocyte/macrophage product that induced collagenase and prostaglandin production in synovial fibroblast cultures [3]. A previous study showed that IL-1 mediated marked downregulation of the matrix metalloproteinases (MMPs) in chondrocytes [4].

It is now generally accepted that chondrocyte senescence and destruction are the final results of abnormal biomechanical factors, and that biochemical and genetic factors also play important roles in the normal functional activities of these cells. MMPs have received much attention because they specifically degrade native collagens and proteoglycans. Levels of MMPs are increased in the cartilage of OA patients, and there is a decrease in levels of tissue inhibitor of metalloproteinase-1 (TIMP-1) [5]. Among these enzymes, MMP-1 and MMP-13 play major roles in degrading the components of the cartilage matrix, especially the aggrecans and collagens. Active stromelysin (MMP-3) also serves as an activator of latent collagenases and digests proteoglycan aggregates in human articular cartilage [6]. TIMPs act as inhibitors of MMPs. TIMP levels are elevated in OA cartilage, possibly reflecting an endogenous adaptive response to the increased levels of active proteinase activity [7].

Nonsteroidal anti-inflammatory drugs (NSAIDs), corticosteroids, and hyaluronan have been clinically used for the treatment of OA in the clinic. However, they fail to reverse cartilage damage, and a proportion of patients still progress and ultimately require surgery. Thus, there is a continuing need for better agents with which to treat OA [8].

Here, we investigated the activity of tetrandrine (Tet) in the protection of articular cartilage. Tet (International Union of Pure and Applied Chemistry name: 6,6′,7, 12-tetramethoxy-2,2′-dimethyl-1*β*-berbaman; Chemical Abstracts Service number 518-34-3; C_38_H_42_N_2_O_6_; molecular weight, 622.74988) is a bisbenzylisoquinoline alkaloid, purified from the root of Stephania tetrandrine of the Menispermaceae family. It has been used as an antihypertensive and antiarrhythmic agent as well as an operative agent in cardiovascular disease because of its calcium channel-blocking effects, and it has been shown to exhibit antifibrotic activity in silicosis [9, 10]. Early findings showed that Tet may have value in the therapy of chronic inflammatory diseases in which IL-1 or TNF play a role in pathogenesis [11, 12]. Recent studies have shown that Wnt/*β*-catenin signalling also participates in the response to mechanical injury to cartilage and that Tet may have an effect on OA through Wnt/*β*-catenin signalling from a study of the effect of Tet on tumour growth in human colorectal cancer [13, 14]. However, little is known about its possible use in the treatment of OA. In this study, we evaluated the effects of Tet on cartilage degradation following intra-articular injection in an experimental OA model and at 20 mg/L in an articular chondrocyte experiment; at this concentration, the effectiveness of Tet in vitro has been confirmed.

## 2. Materials and Methods

### 2.1. Reagents

Tet (Sigma, St. Louis, MO, USA) was dissolved in 0.01 mol/L HCl (1 mg Tet dissolved in 10 uL 0.01 mol/L HCl to mother liquor) and diluted to 20 mg/L by Dulbecco's modified Eagle's medium (DMEM) for the in vivo test and by phosphate-buffered saline (PBS) for the in vitro test.

### 2.2. MTT Assays

Cells were cultured in a 96-well plate (8000/well). After incubation with various concentrations (5–100 mg/L) of Tet for 24 h in a serum-free medium, MTT (5 mg/mL) was added (20 uL/well). Cells were then incubated with MTT for 4 h, and culture medium was removed and DMSO was added (150 mL/well). Absorbance was measured at 570 nm. This step was repeated for three times to get average results to reduce errors.

### 2.3. Primary Cell Culture and Treatment

The study was approved by the Institutional Animal Care and Use Committee of Zhejiang University (Hangzhou, China). Four-week-old New Zealand white rabbits were sacrificed by air embolism. Immediately, cartilage harvested from the knee joints of rabbits under sterile conditions was digested with 0.25% pancreatic enzymes for 30 min to remove other tissues and cells, then digested with 0.2% collagenase II at 37°C for 4 h, as described previously with minor modifications. Cells were grown to confluence in DMEM supplemented with 10% fetal bovine serum (FBS), 100 U/mL penicillin, and 100 mg/mL streptomycin at 37°C with 5% CO_2_. Cells from the third passage were used. Experiments were performed after subconfluent cells were serum-starved overnight. Cells were seeded in six-well plates (1 × 10^5^/well), and subconfluent cells were preincubated with three concentrations of Tet (derived from preliminary tests) for 1 h followed by stimulation with IL-1*β* (10 ng/mL) for 24 h. Cells were then harvested and subjected to determination of MMP-1, MMP-3, MMP-13, and TIMP-1 mRNA expression levels to assess the optimum Tet concentration, which was then used in subsequent experiments.

### 2.4. Gene Expression Analysis

Total RNA was extracted from chondrocytes treated with various Tet concentrations and cartilage harvested from the in vitro test using the TRIzol reagent (Invitrogen, Carlsbad, CA, USA) according to the manufacturer's protocol. Total RNA (600 *μ*g), 1 *μ*L of primer mix, and 1-*μ*L dNTPs (10 mM) were added to a 200-*μ*L RNase-free centrifuge tube. DEPC-treated water was added (15 *μ*L), and the centrifuge tube was incubated on ice. The tube was then incubated at 70°C for 5 min. Next, 4 *μ*L of 5 × first-strand buffer, 2 *μ*L of 0.1 M DTT, 25 units of RNase inhibitor, and 200 units of Superscript II reverse transcriptase (Invitrogen) were added. RNA was reverse-transcribed into cDNA. MMP-1, MMP-3, MMP-13, and TIMP-1 expression levels were quantified by quantitative real-time polymerase chain reaction (qRT-PCR) using the iCycler system (Bio-Rad, Hercules, CA, USA) and iQ SYBR Green Supermix PCR kit (Bio-Rad), based on sequence information (Table 1). A parallel amplification with rabbit 18S primers was carried out to normalise the expression data of the targeted gene transcripts. The relative levels of targeted gene expressions were calculated following the formula:

(1) $$\begin{array}{c} 2^{-(\Delta ct\;\text{target}\;\text{gene}\;-\;\Delta ct\;18\text{s}\;\text{rRNA})}. \end{array}$$

### 2.5. Western Blot Analysis

Cells were rinsed with iced PBS, and total protein was extracted from three samples using a total protein extraction kit, then quantified with a BCA quantification kit. Proteins were resolved by sodium dodecyl sulphate polyacrylamide gel electrophoresis and then transferred onto polyvinylidene fluoride membranes. After blocking for 1 h with 5% milk in Tris-buffered saline-Tween, membranes were incubated with antibodies against MMP-3 (anti-MMP-3 antibody; Santa Cruz sc-6839, USA), TIMP-1 (anti-TIMP1 antibody; Abcam ab126847, USA), *β*-catenin, (anti-beta catenin antibody; Abcam ab83295, USA), and *β*-actin (Santa Cruz Biotechnology) overnight at 4°C. Membranes were incubated with goat anti-mouse IgG-HRP and goat anti-rabbit secondary antibody at room temperature for 1 h, and signals were detected using an Enhanced Chemiluminescence kit (GE Healthcare, Shanghai, China) with exposure to X-ray film (Kodak, Hangzhou, China).

### 2.6. Induction of OA in Rabbits

Fifteen New Zealand white rabbits weighing 2.0 kg were used (Animal Centre of Zhejiang University). All experiments were conducted with the approval of Zhejiang University Animal Care and Use Committee.

Ten of the 15 rabbits underwent bilateral anterior cruciate ligament transections (ACLTs) on the knee joints to induce OA and were divided into two groups randomly. The other five rabbits (control group) received sham operations, which involved opening the articular cavity and resuturing it without cutting the short anterior cruciate ligament. After surgery, all animals were returned to their cages; the limbs were not immobilised. At 1 month after surgery, the Tet group was given intra-articular injections of 0.3 mL Tet (20 mg/L) in both knees once per week for 6 weeks. The OA group was injected with 0.3 mL solvent alone in both knees under the same conditions (solvent: 10 *μ*L of 0.01 mol/L HCl dissolved in 50 mL of PBS). In the sham-operation group, no other procedures were conducted. Rabbits were sacrificed 7days after the last injection.

### 2.7. Histological Examination

Five samples from each group were fixed in 4% paraformaldehyde [15], decalcified with 10% formic acid, buffered at pH 7.4, dehydrated through a series of ethanol solutions, embedded in paraffin, cut into 3-*μ*m sections, and stained with safranin O-fast green. The samples were scored for the degree of histological change using the Mankin score system [16]. Two independent researchers assessed the extent of histological cartilage damage in a blinded manner.

### 2.8. Statistical Analysis

All data were expressed as mean ± standard deviation (SD). Statistical analysis of MTT assay data was performed by an unpaired *t*-test while histological and gene expression data were analyzed by a paired *t*-test. Differences were considered significant at *P* < 0.05.

## 3. Results

### 3.1. Effects of Tet on Viability

**Figure 1:**
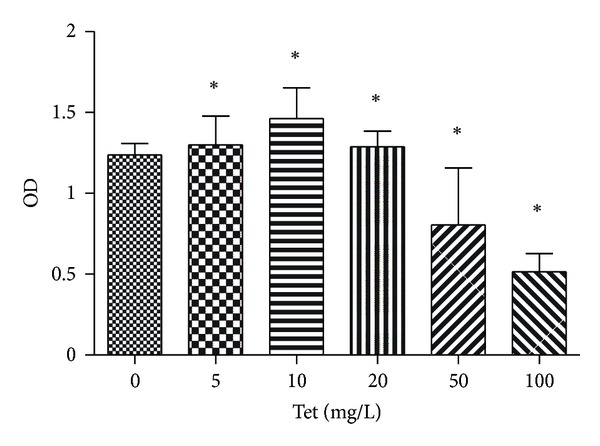
The effects of Tetrandrine (Tet) on chondrocyte proliferation as determined by the MTT assay. Chondrocytes (8,000) were initially plated in each well of a 96-well plate and treated with Tet at 0, 5, 10, 20, 50, 100 mg/L. At 24 h, the number of viable cells was measured by MTT assay. Values are mean ± SD of three independent experiments. *Statistical significance in viable cell numbers (*P* < 0.05).

The chondrocyte toxicities of 100, 50, 20, 10, and 5 mg/L Tet were assessed by MTT assay. Statistical analysis of MTT assay data was conducted using an unpaired *t*-test (Figure 1). Concentrations of >20 mg/L had toxic effects on chondrocytes. As a result, 20 mg/L was selected as the optimum concentration and was used in subsequent experiments.

### 3.2. Effects of Tet on the Expression of MMP-1, MMP-3, MMP-13, TIMP-1, and *β*-catenin in Rabbit Chondrocytes and Cartilage

**Figure 2:**
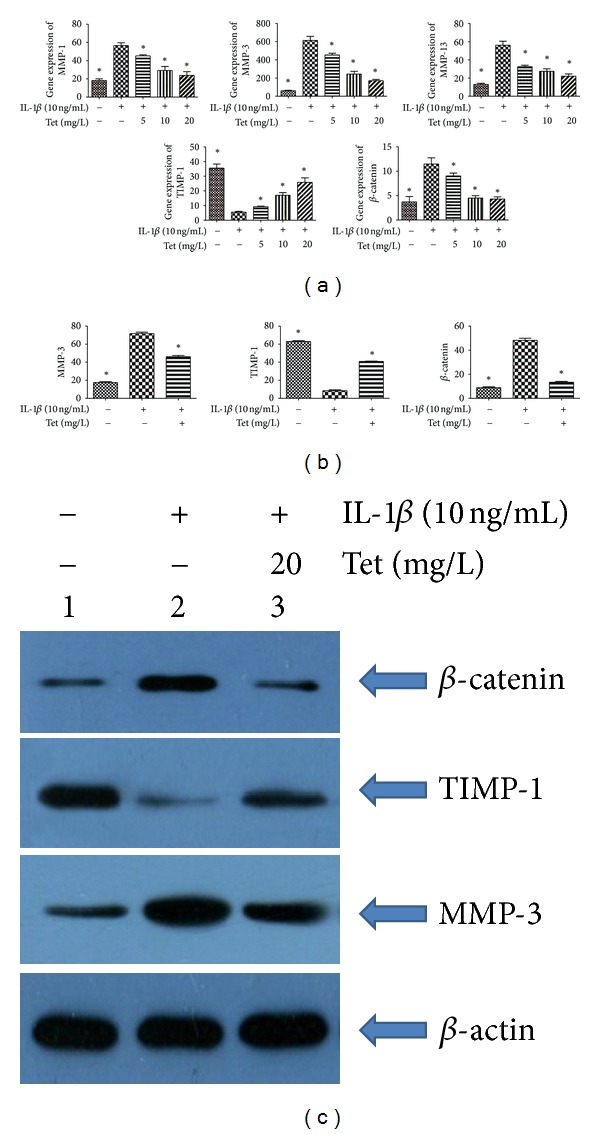
Effects of Tet on the expression of matrix metalloproteinase-1 (MMP-1), MMP-3, MMP-13, tissue inhibitors of metalloproteinase-1 (TIMP-1), and *β*-catenin in chondrocytes. Cells were pretreated with Tet (0 mg/L, 5 mg/L, 10 mg/L, 20 mg/L) for one hour, followed by stimulation with interleukin-1*β* (IL-1*β*) (10 ng/mL) for 24 h. qRT-PCR was performed to determine the expression levels of MMPs in chondrocytes in (a). The protein concentrations of MMP-3, TIMP-1, and *β*-catenin were detected by Western blot analysis with the optimal concentration (b). The protein concentrations of MMP-3, MMP-13, and TIMP-1 were quantitated (c). Data are expressed as mean ± SD. *P < 0.05 compared with cells stimulated with IL-1*β* alone.

**Figure 3:**
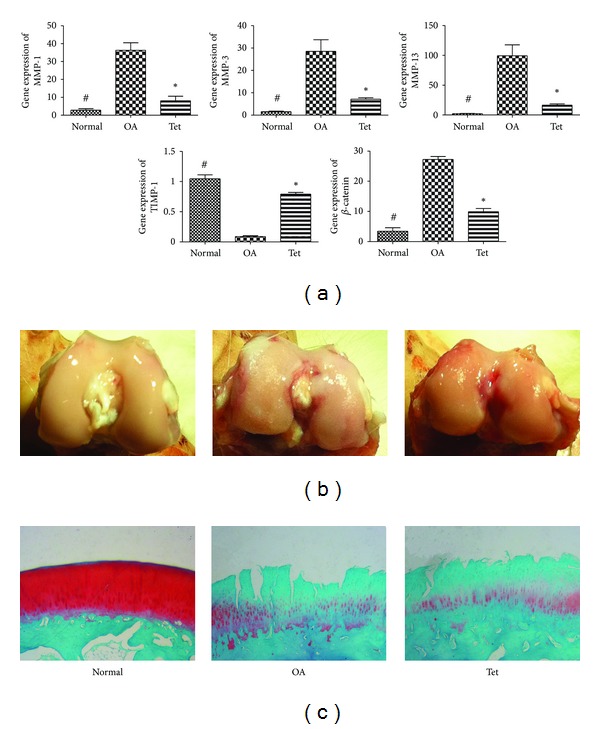
Effects of Tet on the expression of MMP-1, MMP-3, MMP-13, TIMP-1, and *β*-catenin in vivo experiment on cartilage in (a). The Tet and OA groups were treated with ACLT, and only the Tet groupwasgiven treatment of Tet (20 mg/L) for 6 weeks after surgery. Rabbits were sacrificed after last injection in articular cavity. Rabbits receive the sham-operation regarding as the Normal group. Representative pictures of macroscopic observations in (b) and Safranin O staining are shown in (c).

qRT-PCR was performed to determine the expression levels of MMPs in chondrocytes. To investigate the effects of Tet on gene expression, chondrocytes were preincubated with Tet for 1 h prior to stimulation with IL-1*β* for 24 h. Chondrocytes stimulated with IL-1*β* showed induction of MMP-1, MMP-3, and MMP-13 gene expression, but downregulation of TIMP-1 expression (Figure 2(a)). Tet inhibited the IL-1*β*-mediated induction of MMP-1, MMP-3, and MMP-13 gene expression and induced the expression of TIMP-1. We next examined the effects of IL-1*β* and Tet on protein expression of MMP-1, MMP-3, MMP-13, and TIMP-1 in chondrocytes. The optimum Tet concentration was used (Figures 2(b) and 2(c)). Treatment with IL-1*β* resulted in the upregulation of MMP-1, MMP-3, and MMP-13 and the down-regulation of TIMP-1 at the protein level. These effects were blocked by Tet. We identified similar changes in the analysis of the expression of the MMP, TIMP-1, and *β*-catenin genes in vivo (Figure 3(a)).

### 3.3. Macroscopic Observations

In the sham-operation (control) group, the cartilage on the femoral condyles was macroscopically normal, with a smooth, glistening surface, and no cartilage defect or osteophyte was observed. In the OA group, general characteristics of OA, including erosion and osteophyte formation, were seen on the side of the femoral condyles after surgery. The Tet group showed less bone wear than the OA group as determined by gross appearance (Figure 3(b)).

### 3.4. Histopathological Changes in Articular Cartilage

**Figure 4:**
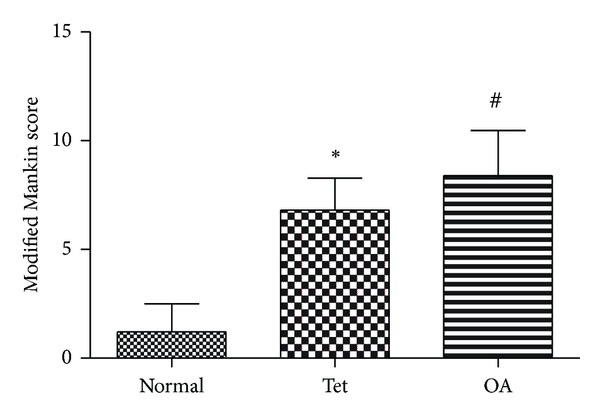
The Mankin scores are also presented. ^#^ *P* < 0.05 when Normal group compared with OA group; *P < 0.05 when Normal group compared with Tet group.

Histopathological changes in the rabbits after surgery centred mainly on the thinner cartilage layer, abraded surface, and reduced safranin O-fast green staining in the cartilage. Tet inhibited the cartilage degradation, which developed as OA progressed. However, osteophyte proliferation could not be reversed (Figure 3(c)). Consistent with these findings, the Mankin score was reduced in the Tet group compared with the OA group (Table 2 and Figure 4).

## 4. Discussion

OA may be of unknown origin (idiopathic, primary) or related to a known medical condition or event; the major pathological changes occur in the structure of the hyaline cartilage, with a variable degree of synovial inflammation. These changes are ascribed to a complex network of biochemical factors, including proteolytic enzymes, matrix metalloproteinases, and cytokines, which interact and lead to the breakdown of cartilage macromolecules. Cytokines, such as TNF-*α* and IL-1 produced by mononuclear cells, activated synoviocytes, or even articular cartilage itself, significantly upregulate MMP gene expression [17]. As a result, cytokines affect compensatory chondrocyte synthesis pathways, leading to the degradation of the extracellular matrix (ECM). Many studies have demonstrated that IL-1 and TNF-*α* inhibit chondrocyte compensatory biosynthesis pathways, which can further compromise cartilage repair [18]. IL-1*β* is known to play a pivotal role in cartilage degradation, through the induction of MMPs secreted by chondrocytes. Chondrocytes stimulated with IL-1*β* in vitro have been used to mimic the microenvironment that occurs in OA [19].

In this in vitro study, we used IL-1*β* to induce MMP gene expression, based on the hypothesis above, then evaluated the effect of Tet on MMP induction in rabbit chondrocytes, as well as its effects on ACLT-induced OA in the rabbit model [20]. We demonstrated that Tet inhibited the IL-1*β*-mediated induction of MMP-1, MMP-3, and MMP-13 and induced the expression of TIMP-1, a metalloproteinase inhibitor, in rabbit articular chondrocytes; these effects were manifested at both the mRNA and protein levels. The MMPs are a family of proteinases that are involved in ECM degradation. MMP-1 and MMP-13, in particular, are interstitial collagenases that degrade type II collagen in cartilage, a major component of the ECM, which is a committed step in the progression of OA. As a result, targeting MMPs represents a promising approach to the treatment of OA. MMPs can be induced by IL-1*β*, and increased expression of MMPs has been identified in osteoarthritic cartilage [21]. Similarly, serum concentrations of MMP-3 are elevated in patients with OA compared with normal subjects [22].

In the present study, pretreatment with 20 mg/L Tet resulted in significantly decreased expression levels of MMP-1, MMP-3, and MMP-13 in IL-1*β*-induced chondrocytes. Tet also induced the expression of TIMP-1 without causing cytotoxicity. Because TIMPs are endogenous inhibitors of MMPs, we suggest that Tet may modulate the balance between MMP and TIMP expression to exert its antiarthritic effects in IL-1*β*-treated rabbit articular chondrocytes [23]. We also investigated in vivo the effects of Tet on cartilage degradation in an ACLT-based experimental model of OA in rabbits. This model has been widely used to evaluate the efficacy of agents in the treatment of OA due to the model's mechanical instability, which leads to cartilage degradation [24]. As shown in this study, ACLT in the rabbit resulted in cartilage degradation, and injection of Tet into the articular cavity (0.3 mL, 20 mg/L) for 6weeks inhibited cartilage degradation as assessed by histological evaluation. The results were consistent with the in vitro findings. These observations provide evidence that Tet possesses chondroprotective activity both in vitro and in vivo.

Several signalling pathways are involved in the induction of MMPs by IL-1*β* in chondrocytes. Among them, the Wnt/*β*-catenin signalling pathway and proteins regulate organ development, tumourigenesis, and bone homeostasis, among other functions, and are believed to play important roles in the induction of MMP expression [25, 26]. Given this, agents that interfere with the Wnt/*β*-catenin signalling pathway may affect MMP expression. In the present study, we investigated whether Tet affected activation depending on the Wnt/*β*-catenin signalling pathway. *β*-catenin is a multifunctional protein, involved in the formation of adhesive “tape” by interacting with cadherin at cell junctions. Furthermore, free *β*-catenin can penetrate into the nucleus to affect gene expression [27].

Our in vitro and in vivo findings suggest that expression of MMP and *β*-catenin genes showed a similar trend, as did the protein levels. We demonstrated that Tet effectively targets the Wnt/*β*-catenin signalling pathway in chondrocytes, suggesting that inhibition of the Wnt/*β*-catenin signalling pathway may, at least in part, account for changes in the trends in the expression of MMPs and TIMP-1, as well as cartilage protection. This study is, to our knowledge, the first to report the effects of Tet on Wnt/*β*-catenin signalling in chondrocytes and in an ACLT-based experimental model of OA in rabbits. These findings are consistent with previous studies of the effects of Tet on the Wnt/*β*-catenin signalling pathway in other cell systems [14]. The discrepancies between our findings and these reports may be related to differences in cell type and stimulation conditions.

In conclusion, modulation of cytokines, such as IL-1*β* and TNF-*α*, which control MMP gene overexpression, appears to be a fertile target for drug development for the treatment of OA. Several studies have illustrated the potential importance of modulating IL-1 activity as a means to reduce the progression of structural changes in OA [24]. In the present study, we demonstrated that Tet possessed chondroprotective effects in IL-1*β*-induced rabbit chondrocytes and an experimental model of OA. The inhibition of MMPs by Tet was associated at least in part with inhibition of the Wnt/*β*-catenin signalling pathway. Our results indicate that Tet shows promise as a therapeutic agent for the treatment of OA. However, further studies are needed to confirm and extend these preliminary findings.

## Figures and Tables

**Table 1:** Primers of targeted genes.

Gene	Genbank accession	Primer sequences(5′ to 3′)	Size (bp)	Annealing (°C)
Rabbit MMP-1	M25663	F: CAGGAGCCTTCCCAAGAGGAA	79	62
		R: CTTGTCTCTTGCATATCAGGATGATG		
Rabbit MMP-3	NM_001082280	F: ACACCGGATCTGCCAAGAGA	89	63
		R: CTGGAGAACGTGAGTGGAGTCA		
Rabbit MMP-13	NM_001082037	F: CAGATGGGCATATCCCTCTAAGAA	88	63
		R: CCATGACCAAATCTACAGTCCTCAC		
Rabbit TIMP-1	AY829730	F: CAACTGCGGAACGGGCTCTTG	102	63
		R: CGGCAGCGTAGGTCTTGGTGAA		
Rabbit *β*-catenin	DQ786777	F: CGTACGCACCATGCAGAACACAAA	154	63
		R: ATCCACTGGTGAACCGAGCATCTT		
Rabbit 18S	EU236696	F: GACGGACCAGAGCGAAAGC	119	62
		R: CGCCAGTCGGCATCGTTTATG		

**Table 2:** Histological score of articular cartilage.

Femoral condyle	Normal group	Tet group	OA group
Structural changes	0.2 ± 0.40	3.0 ± 0.63*	3.8 ± 1.17^#^
Cellular changes	0.4 ± 0.49	1.6 ± 0.80*	1.8 ± 0.75^#^
Safranin staining	0.4 ± 0.49	1.8 ± 0.75*	2.2 ± 0.75^#^
Tide mark	0.2 ± 0.4	0.4 ± 0.49*	0.6 ± 0.49^#^
Sum of score	1.2 ± 1.17	6.8 ± 1.33*	8.4 ± 1.85^#^

Values are the means ± SD. ^#^ *P* < 0.05 when Normal group compared with OA group, *P < 0.05 when Normal group compared with Tet group.
